# Clinical presentation and molecular diagnosis of a possible Mpox virus and Varicella zoster virus co-infection in an adult immunocompetent Filipino: a case report

**DOI:** 10.3389/fpubh.2024.1387636

**Published:** 2024-11-21

**Authors:** April Keith Balingit, Phoebe Grace Grande, Amalea Dulcene Nicolasora, Francisco Gerardo Polotan, Roslind Anne Pantoni, Miguel Francisco Abulencia, Maria Yna Joyce Chu, Nicole Rivera, Marie Socouer Oblepias, Jemelyn Garcia

**Affiliations:** Research Institute for Tropical Medicine, Muntinlupa, Philippines

**Keywords:** rash, Mpox, Varicella zoster, co-infection, Philippines

## Abstract

We report the first travel-related case of a possible Mpox-Varicella zoster virus (VZV) co-infection in the Philippines, a country that is endemic for Varicella but non-endemic for Mpox. A 29-year-old Filipino, female, with a travel history to Switzerland and with no prior history of VZV infection sought consultation due to rashes. She presented with multiple papular, pustular, and vesicular skin lesions, some with umbilication and with irregular borders, on the face, neck, trunk, inguinal area, upper extremities, and right leg. She also had bilateral submandibular and post-auricular lymphadenopathies. Tzanck smear exhibited viral cytopathic effects. She tested positive for Mpox infection (Clade II) and Varicella infection via quantitative real-time polymerase chain reaction (qPCR) tests but with a high CT value obtained from the Mpox PCR. Shotgun metagenomic sequencing (mNGS) successfully recovered sequences from the Varicella zoster virus which corroborated with the high viral load detected using qPCR. In contrast, shotgun mNGS was not able to generate a Mpox consensus sequence due to very few reads mapped to the Mpox virus reference sequence, which raised the question if there was the presence of a true Mpox-Varicella co-infection in our patient. Nevertheless, systemic and topical acyclovir was given to the patient. She was discharged and continued home isolation for 30 days from the rash onset. Strategies have been formed by the country’s healthcare facilities to properly identify Mpox infection. However, Mpox co-infection with other viral diseases presented a challenge in the proper diagnosis of our patient. This prompted a high index of suspicion and the usage of suitable diagnostic tests. With proper clinical evaluation and utilization of appropriate diagnostic tests, we were able to diagnose the first Filipino patient with a possible Mpox and Varicella zoster virus co-infection.

## 1 Introduction

Mpox (formerly Monkeypox) is a re-emerging infectious disease known to be endemic in Central and West Africa but unexpectedly created new outbreaks worldwide in May 2022. It is caused by the Mpox virus (MPXV), an Orthopoxvirus in the *Poxviridae* family, and is acquired via zoonotic and human-to-human transmission through respiratory secretions or direct contact with skin lesions of infected animals or individuals (1). The classic Mpox rash usually starts from macular lesions developing into papules and pustules which eventually form central umbilication and then crusts (2). The rash is usually accompanied by lymphadenopathy and is preceded by a prodromal period. A Mpox patient is considered infectious until all scabs have fallen off. Meanwhile, reports from the recent global Mpox outbreak described infected patients who presented with atypical symptoms and rash characteristics (3).

There are currently around 65,000 Mpox cases reported worldwide, with four cases found in the Philippines at the time of writing this manuscript (4). Following the detection of Mpox cases in Filipinos, guidelines on Mpox diagnosis, treatment, and prevention have been strengthened to help healthcare providers in differentiating it from other disease conditions with similar clinical presentation. One of these is chickenpox caused by the Varicella zoster virus (VZV). In regions of the world where both viruses are present, there is confusion in the diagnosis of Mpox and VZV (5). VZV is also a DNA virus-like Mpox, but it belongs to the *Herpesviridae* family and is only transmitted among humans (6). Contrary to Mpox, the typical Chickenpox rash presents simultaneously at different stages on the skin, with lymphadenopathy being an uncommon occurrence and the appearance of fever more commonly seen before or during rash onset (6). VZV is contagious beginning one to two days before rash onset until all lesions have crusted. It is known to occur worldwide, but it is mostly seen in children living in temperate regions and in adults living in tropical countries such as the Philippines (7). Without knowing these key characteristics, Mpox is often misdiagnosed for VZV or vice versa. Moreover, cases of co-infections of the two viruses have been reported by surveillance studies in Africa (8). Case reports on Mpox-VZV co-infections in Brazil have also been published which comprehensively described the presentation of each case (9). We then report the first travel-related case of a possible Mpox-VZV co-infection in the Philippines, a country that is endemic for Chickenpox but non-endemic for Mpox.

## 2 Case presentation

We present a case of a 29-year-old, female, Filipino, who consulted due to multiple pustular and vesicular rashes on the face, neck, trunk, inguinal area, bilateral upper extremities, and right leg. The patient had no known comorbidities, no history of varicella or measles infection, and no known allergies to food or drugs. She claimed to have a complete primary childhood immunization under the Philippine National Immunization Program, which included a Varicella zoster vaccine, but was unable to provide her vaccination record during the time of consultation. She was a nonsmoker and an occasional alcoholic beverage drinker. She denied use of illicit drugs. She only had one long-term male sexual partner. Patient denied having sexual relations with any other person aside from her partner.

**Figure 1:**
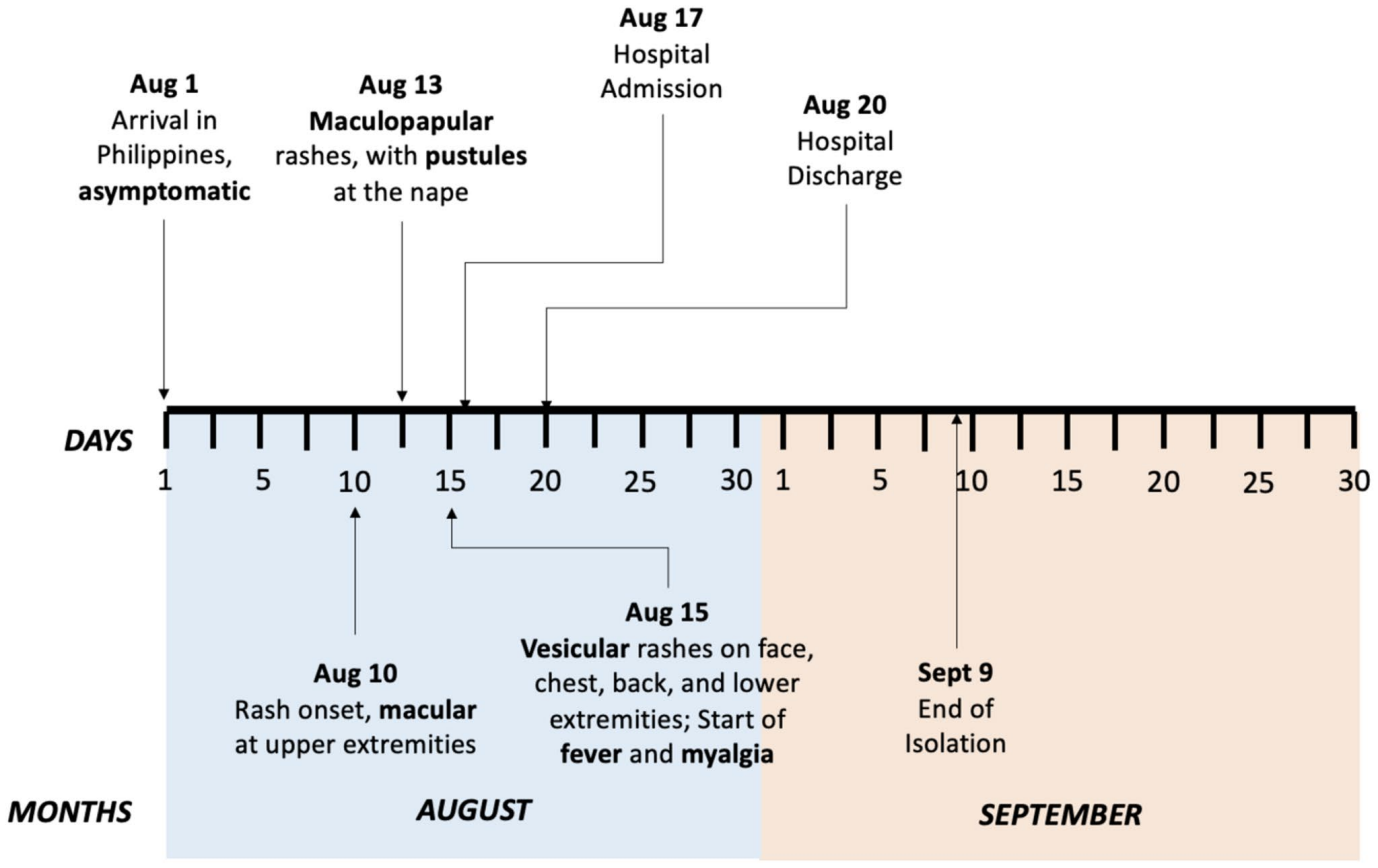
Timeline of patient’s symptom progression to end of isolation, August to September 2022.

Travel history revealed the patient’s work-related trip to Geneva, Switzerland from February to July 2022. She did not visit any other countries during her stay in Geneva. She made use of public transportation, mainly buses and trains, to go to work daily. She left Geneva on July 31, 2022, and arrived in the Philippines on August 1, 2022, with no reported symptoms. Ten days after her arrival, she noticed small pruritic macular rashes erupting on both of her arms. She did not seek medical consultation nor received any intervention. Thirteen days after her arrival, she noted an increase in the number of her skin lesions which progressed to maculopapular rashes. She also noted the appearance of an erythematous pustule on her nape. No other associated signs and symptoms were noted. Fifteen days after her arrival, her skin lesions progressed to vesicular pruritic rashes on her face, chest, back, and lower extremities with accompanying undocumented fever and myalgia. Sixteen days after arrival, she went for a consultation at a local clinic due to the persistence of her rashes. She was advised to contact the city health office which referred her to a local hospital for evaluation. Figure 1 shows the timeline of the patient’s symptom progression.

**Figure 2:**
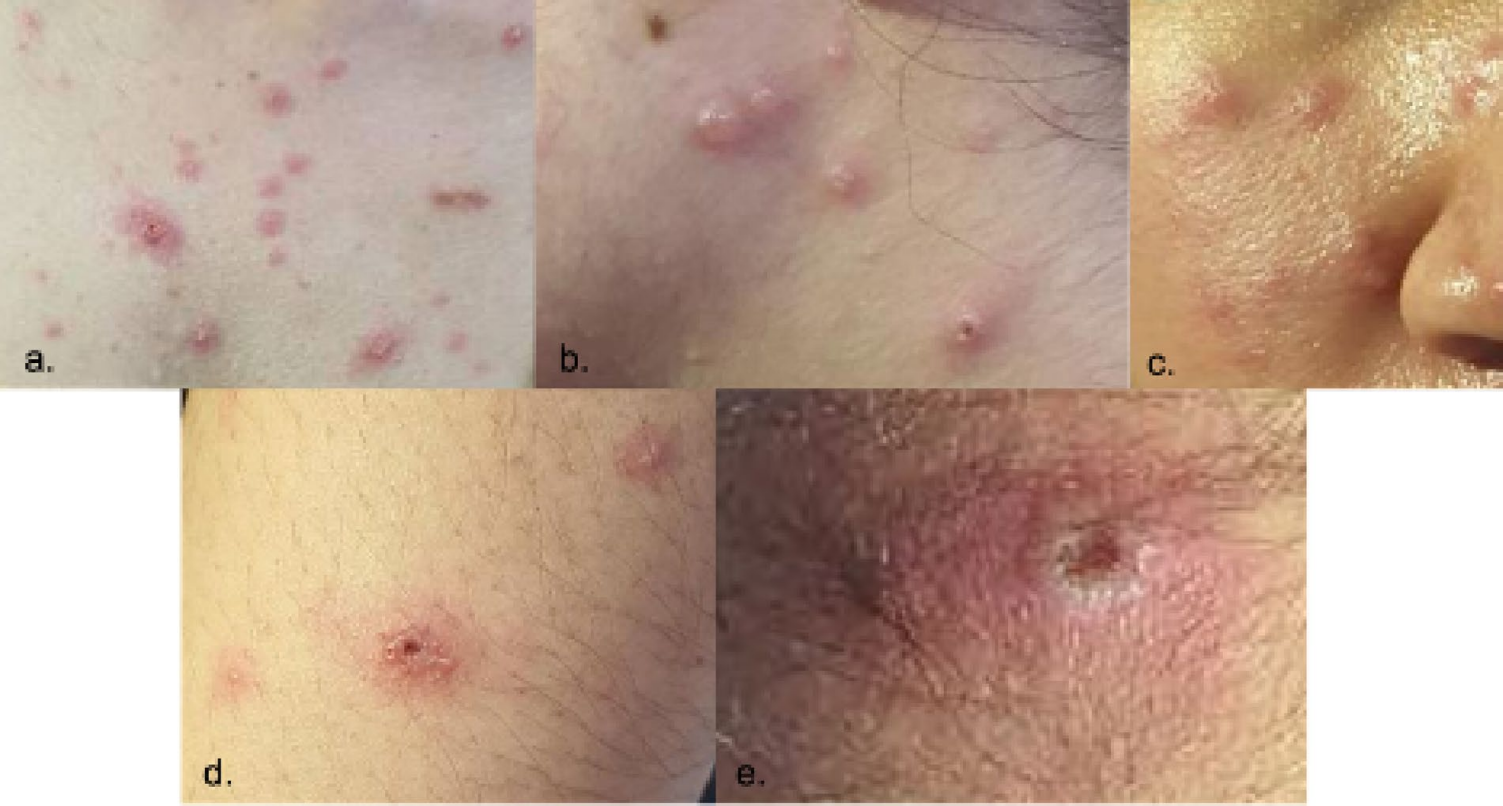
Skin lesions presenting at different stages upon admission (Day 7 from rash onset). Locations: (a) chest, (b) neck, (c) right cheek, (d) left arm, (e) nape.

The patient was seen at the emergency room with blood pressure of 100/70 mmHg, heart rate of 89 beats/min, respiratory rate of 20 cycles/min, body temperature of 38.1°C, and oxygen saturation of 99% at room air. Pertinent physical examination findings were multiple papular, pustular and vesicular skin lesions, some with umbilication, some with irregular borders, presenting at different stages on the face, neck, trunk, inguinal area, bilateral upper extremities and right leg. She also had bilateral submandibular and post-auricular lymphadenopathies (Figure 2). Other physical examination findings were unremarkable.

Following the local guidelines for screening patients presenting with rashes at the emergency room, she satisfied the criteria for Mpox Suspect hence she was admitted in the isolation room for further evaluation. VZV infection was also considered due to the presentation of skin lesions at different stages of development. Initial blood tests showed a white blood cell count of 4.5 × 10^9^/L, neutrophils 75%, lymphocytes 15%, and a normal urinalysis result. Measles infection was ruled out with a negative measles polymerase chain reaction (PCR) test result. HIV screening was also done which showed a non reactive result. No further laboratory tests for sexually transmitted infections (syphilis, hepatitis, chlamydia, and gonorrhea specifically) were done for the patient.

**Figure 3:**
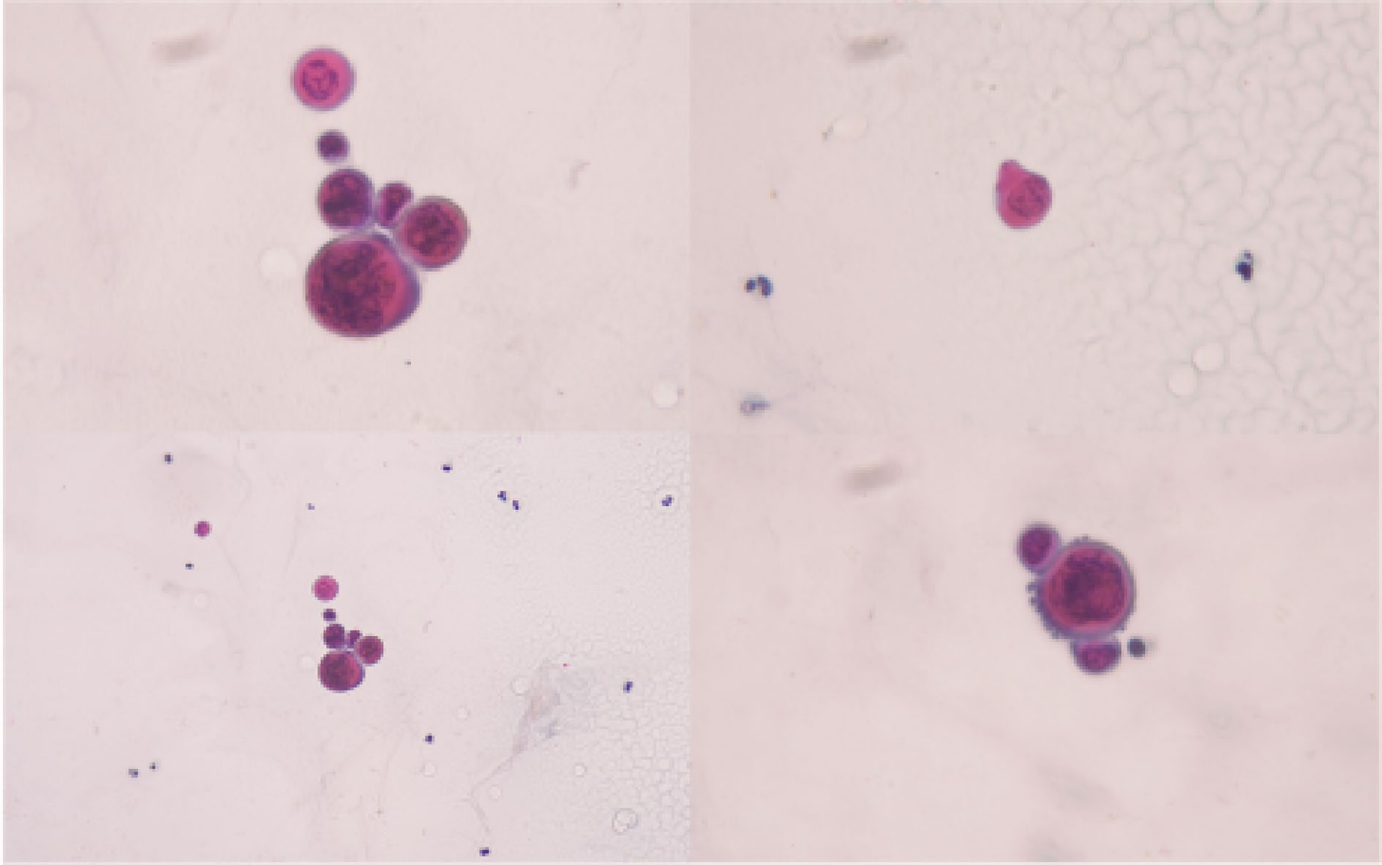
Tzanck smear of the vesicular skin lesions. Tzanck smear of the patient’s vesicular skin lesion shows neutrophils with atypical round cells suspected to exhibit viral cytopathic effects.

The patient was referred to the Dermatology service who conducted a Tzanck smear test which showed neutrophils with rare atypical round cells exhibiting viral cytopathic effects, suggesting a viral etiology (Figure 3).

The patient’s plasma tested positive for VZV using a real-time quantitative polymerase chain reaction (qPCR) test with 5,350 copies/mL detected. Meanwhile, a total of nine specimens (three samples of skin scrapings and six vesicle fluid swabs) were obtained and sent to the Special Pathogens Laboratory for a confirmatory probe-based Mpox qPCR test. Nucleic acid extraction from dry swab and tissue samples were performed using QiaAmp DNA Mini Kit (QIAgen, Hilden, Germany, Cat No: 51306) according to the manufacturer’s instructions (10). The PCR primers and probes were developed from the sequences described by Li et al. (11) (Supplementary Table S1). Probe-based real time PCR assay was performed using Applied Biosystem’s AgPath-ID One Step PCR kit (4387424) (12) and Bio-Rad CFX96 Touch real time PCR machine as PCR platform. RNase P was the assays’ internal target control.

One lesion dry swab and one lesion roof specimens were confirmed to be positive for Mpox RT-PCR with a mean cycle threshold (Ct) value of 36.20 (Supplementary Table S2), indicating a low viral load. The Mpox RT-PCR differentiation assay also revealed that the same samples belong to Mpox Clade II (previously known as the Western African clade) with mean Ct value of 35.62 (Supplementary Table S2). No viral copies of the Congo Basin clade were detected via RT-PCR among all the samples.

The PCR-confirmed samples from the patient were endorsed to the Molecular Biology Laboratory for genetic characterization by shotgun metagenomic sequencing (mNGS) using the Illumina DNA Prep kit and the Illumina MiSeq instrument. Analysis of the recovered sequences from shotgun mNGS showed too few reads mapped to the Mpox virus reference sequence, hence, we were not able to generate a concensus Mpox sequence for this case. Further analysis showed recovery of a relatively large number of sequencing reads (*n* = 280,805) aligning to *Human alphaherpesvirus* 3 or commonly known as Var*icella zoster* virus (VZV) (Table 1), further confirming the presence of a true VZV infection.

**Table 1:** Metagenomic sequencing reads mapped to *Human alphaherpesvirus* 3.

Sample ID	*Human alphaherpesvirus* 3 (mapped sequencing reads)	Lineage
MPOX22-00061DSA Lesion surface dry swab	40,662	Viruses>Herpesvirales>Herpesviridae>Alphaherpesvirinae>Varicellovirus
MPOX22-00061RA Lesion roof	240,143	
MPOX22-000061 Total Number of reads	280,805	

**Figure 4:**
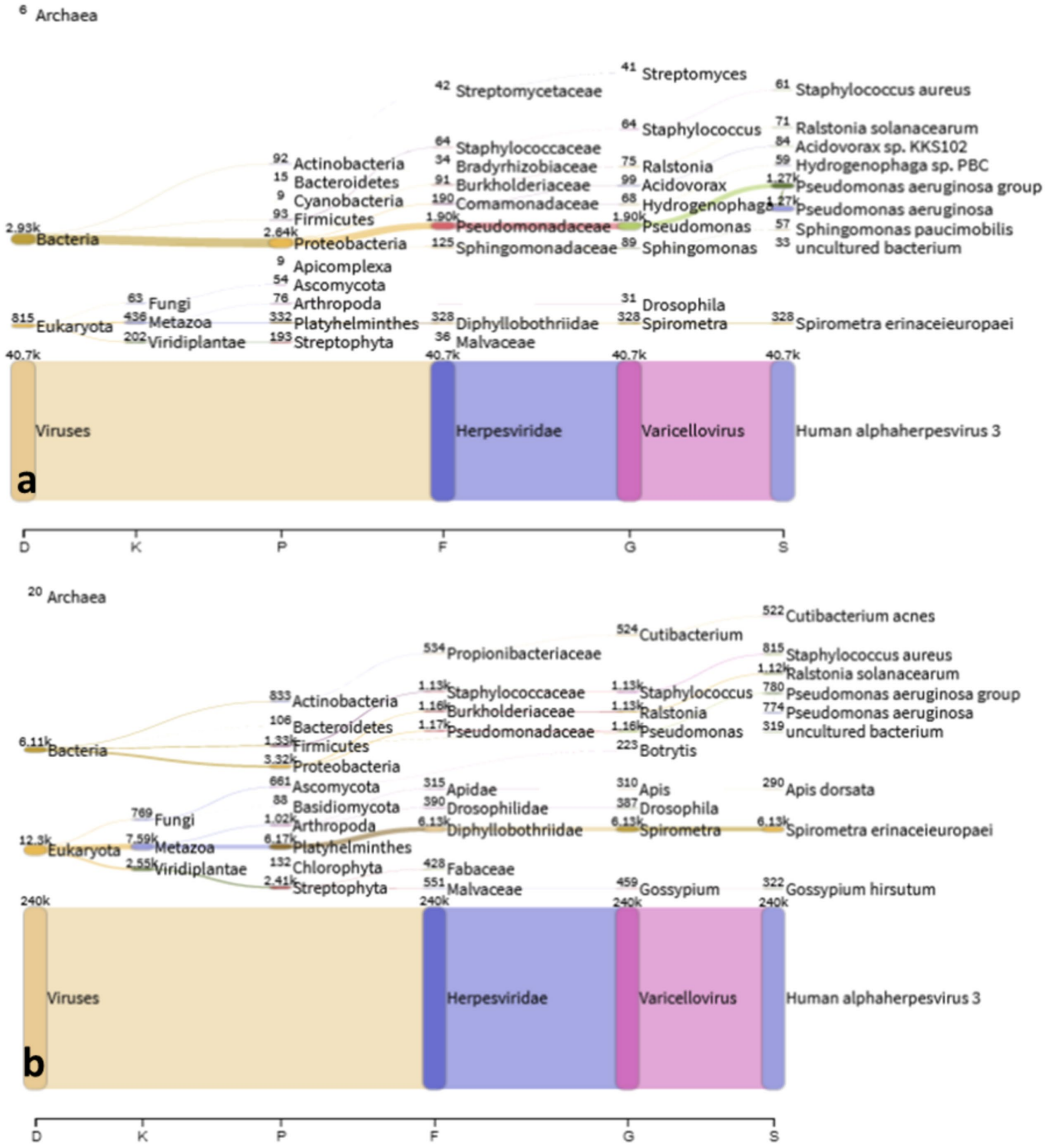
Sankey plot. Classified viral and microbial taxa from MPOX22-0061DSA (a) and MPOX22-0061RA (b) metagenomic samples.

Figure 4 shows the identified microbial and viral taxa from the metagenomic sequences of MPOX22-00061DSA (Figure 4A) and MPOX-00061RA (Figure 4B) samples as depicted by Sankey diagrams, which show the counts of paired-end reads assigned to a particular taxon as indicated by the number on the upper left corner of the taxon. The diagrams show that the majority of the viral sequences recovered from the lesion specimens aligned to the *Human alphaherpesvirus* 3, supporting the high viral infection detected from the patient serum through RT-PCR testing. No reads assigned to Mpox virus were detected in the metagenomic sequences, which was found with low viral load in RT-PCR. Additional reads for the *Pseudomonas aeruginosa* group in the MPOX22-0061DSA sample and for *Spirometra erinaceieuropaei* and *Ralstonia solanacearum* in the MPOX-00061RA sample were also obtained. Since shotgun metagenomic sequencing was used, reads from all organisms present in the sample were obtained. The detected bacterial organisms and other taxa with very low read counts may be considered misassigned taxa, contaminants, or otherwise members of the normal host tissue microflora. Regardless, additional analysis and filtering are needed to draw a definite conclusion.

**Figure 5:**
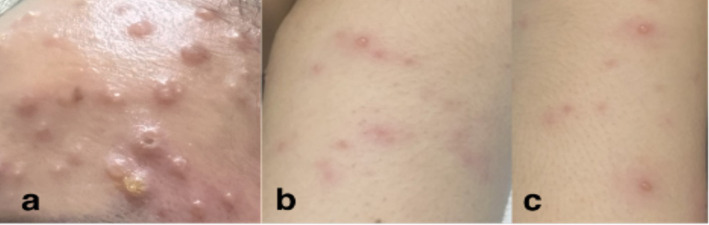
Skin lesions on Day 17 from rash onset. Locations: (a) forehead, (b) left arm, (c) right leg.

**Figure 6:**
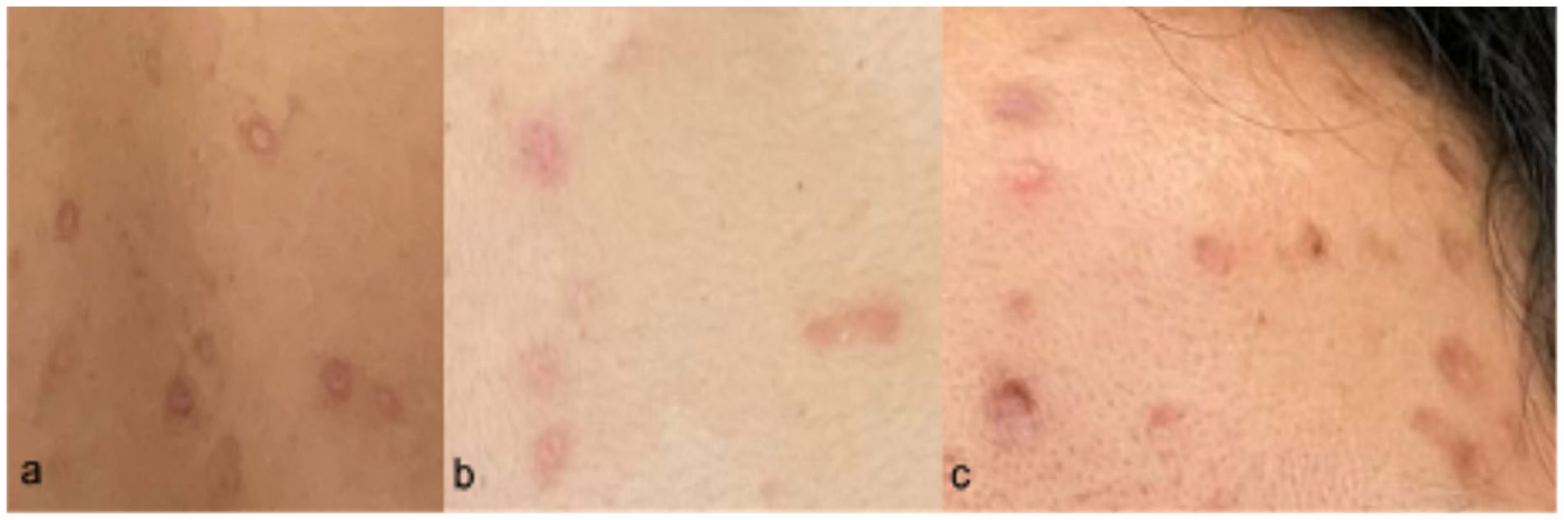
Skin lesions on Day 30 from rash onset. Locations: (a) back, (b) chest, (c) forehead.

The patient was fully advised regarding her disease conditions, and she was started on Acyclovir 800 mg/capsule 1 capsule five times a day for five days accompanied by Acyclovir + Zinc oxide ointment 50 mg/100 mg twice a day to treat the active VZV infection. Mupirocin ointment was also applied to eroded areas. On the second hospital day, the patient had a low grade fever of 37.9°C with the appearance of new pustules, papules, and vesicles on the face, chest, back, and palms. She was discharged on the fourth hospital day in stable condition, with no recurrence of fever for 24 hours, and with good prognosis. She was advised on continued isolation at home until all crusts and scabs have completely disappeared. Home isolation and daily monitoring of symptoms and rash progression or resolution were done via teleconsultations (Figure 5). The patient’s total home isolation lasted 30 days from the date of rash onset. There was no serious complication during the course of her illness (Figure 6). Local contact tracing was done by the local surveillance agencies and none of her close contacts developed the disease symptoms within the observation period.

## 3 Discussion

This report describes the first documented case of a possible Mpox-VZV co-infection in the Philippines in a female patient with significant travel history to Switzerland, who has no prior history of VZV infection. The case is unique because Mpox infection, specifically from clade IIb, are rarely seen in women and women usually contract the virus via sexual transmission. Our patient presented with vesiculo-pustular lesions and tested positive for both Mpox and Varicella viral infections using a quantitative real-time polymerase chain reaction (qPCR) test. This raises the question on whether or not there exists a true viral co-infection in the patient.

There are a few studies that describe and explain the occurrence of Mpox-VZV co-infection in humans, and many of them are surveillance studies carried out in African nations where Mpox is endemic. A previous study conducted in the Democratic Republic of Congo showed that Mpox-VZV co-infection occurred in 13% of the study population and in 19.3% of those who had laboratory-confirmed Mpox infection (8). Mechanisms explaining the occurrence of this phenomenon remain unknown but previous studies suggested that prior infection with either Mpox or VZV may make the host susceptible to acquiring a secondary infection (8). A break on the skin also becomes an ideal point of entry for Mpox via direct contact with infected animals or humans. Moreover, the presence of both viruses in the same host prompted theories from previous studies that acute Mpox infection somehow activates latent VZV infection leading to shingles (13, 14). Whether or not the co-occurrence of the two viruses in a single host is a coincidence or not, further evidence is still required to prove their association.

Overlapping clinical features of Mpox and VZV infections were appreciated in this case, which has not been reported in the local setting. A few surveillance studies in the Democratic Republic of Congo previously investigated cases of Mpox-VZV co-infection and results showed a higher burden of skin lesions found in patients with Mpox-VZV co-infection than VZV infection alone and a lower burden of skin lesions than Mpox infection alone, which suggested the possibility of the two viruses modulating the severity of the infection (8). It is also important to be familiar with the classic presentation of both Mpox and VZV infections for proper diagnosis, especially in countries where both viruses are found to be naturally occurring. The centrally umbilicated pustular lesions with accompanying bilateral lymphadenopathies observed in the case are consistent with the classic Mpox infection as described in previous studies (5). The typical Mpox infection usually has a centrifugal pattern of lesion distribution, with most of the lesions observed at the face, and upper and lower extremities which were also observed in our patient. The recent 2022 Mpox outbreak also reported anogenital rashes among patients in non-endemic countries (15). On the other hand, the patient was also observed to have lesions that were at different stages as well as lesions on the trunk which are more commonly seen with VZV infection (5, 16). Interestingly, the patient’s fever was seen to have occurred after rash onset which was not commonly observed in patients with Mpox nor VZV infection (5). With the recent 2022 Mpox outbreak in multiple non-endemic countries, the need for updated diagnostic pathways arises to differentiate Mpox infection from VZV infection and to determine the presence of possible co-infections.

Tzanck smear was performed in this case since Varicella infection was considered. The result was consistent with a viral etiology showing neutrophils with rare, atypical round cells exhibiting viral cytopathic effects (Figure 2). However, a Tzanck smear alone does not distinguish a Mpox infection from other herpetic infections (17). The gold standard in diagnosing both Mpox and VZV infection involves qPCR tests which were used to diagnose the patient presented in this case (6, 18, 19). For qPCR of Mpox samples, the recommended types of specimens are swabs of skin lesions with or without exudates, roofs, or crusts from more than one lesion (18). On the other hand, fluid or scabs from vesicular lesions are collected for VZV PCR (6). Plasma was used for Varicella PCR in this case. Although plasma and serum specimens are not usually used for VZV PCR tests, previous studies showed their role in the diagnosis and management of VZV infection (20–22).

Sequencing can be performed for further genetic characterization of PCR-positive samples. Metagenomic sequencing (mNGS) is a preferable tool for detecting multiple pathogens present in a sample. Shotgun metagenomic sequencing is a hypothesis-free or untargeted (no pathogen target) sequencing method that allows for the sequencing of all microbial genomes. This sequencing method has been widely used to detect the Mpox virus, other unknown pathogens, and pathogen co-infections. However, mNGS requires high viral concentrations to be able to recover pathogen sequences. In this case, the patient sample demonstrated a high Ct value in the Mpox PCR assay denoting a low viral load of the Mpox virus. High CT value simply means that more reaction cycles are needed to detect a viral RNA and it is commonly observed when there are only trace amounts of viral RNA in the specimen. There is a possibility that our case patient has true Mpox infection but there was low abundance of MPXV in the specimen which may resulted to having no reads in the metagenomic sequencing test. In considering the presence of a true Mpox infection in our patient, we also have to take into consideration that the G2R_G assay that we used in our institute for Mpox detection during the 2022 Mpox outbreak was reported to give false positive results in some specimens (23). For specimens with high CT values (~34 or higher), the Centers for Disease Control and Prevention (CDC) recommends to do an immediate re-extraction and re-testing to ensure that there will be no cross-contamination (23). Unfortunately, our team was not able to do re-extraction and re-testing for Mpox in our patient to confirm any presence of cross-contamination. Hence, there still exists a possibility that our patient might have false positive PCR result for Mpox.

Meanwhile, VZV PCR assay detected a high viral load (5,350 copies/mL) of Varicella zoster virus. The VZV PCR results strongly suggests the presence of a varicella infection in the patient. Relative to the MPXV and VZV viral loads of the specimens obtained from the patient, the sequencing results corroborate the PCR results wherein no reads were recovered for Mpox while a large number of reads were recovered for varicella. The absence of Mpox genome sequence recovery may be due to the low viral load of Mpox present in the patient sample or the patient is not infected with Mpox at all. Previous work on VZV-MPXV co-infection have also reported that the two viruses form separately localized lesions, wherein no lesion with detected VZV was found simultaneously positive for Mpox, or *vice-versa* (8). The sampled specimen from the patient subjected to mNGS may have thus only contained Varicella and not Mpox virus. Finally, the positive results for detecting Mpox across replicates of RT-PCR tests (Supplementary Table S2) are more consistent with a possible Varicella-Mpox co-infection rather than with false positive RT-PCR results. It would require re-sampling and re-testing to confirm the existence of a true Mpox-Varicella co-infection.

Strict isolation and supportive management of symptoms remain central to the management of both Mpox and VZV infections (18). There are currently no available antiviral medications for Mpox infection; however, Acyclovir remains one of the approved drugs for the treatment of early VZV infection (24). Local guidelines recommend that confirmed Mpox patients undergo isolation until symptoms have resolved and until all scabs are gone (18). On the other hand, patients with confirmed Varicella infection are advised isolation until all lesions have crusted (25). The patient reported in this case underwent isolation for 30 days from the occurrence of her rashes until all scabs had disappeared. No serious complication was observed during her home isolation. She was treated for VZV infection with oral and topical Acyclovir for five days. No other systemic antibiotics or antivirals were used by the patient.

## 4 Conclusion

Strategies have been formed by the country’s healthcare facilities to properly identify Mpox infection and differentiate it from other infectious diseases. However, Mpox co-infection with other viral diseases, specifically Varicella zoster infection, clinically presented a challenge in the proper diagnosis of our patient. It is unusual for different infectious agents to cause comparable diseases and circulate in the same population. This prompted a high index of suspicion and the usage of suitable diagnostic tests. Tzanck smear could not differentiate between Mpox and Varicella zoster viruses. Real-time quantitative polymerase chain reaction (qPCR) tests confirmed the presence of both Mpox and Varicella zoster virus infections in the patient but the true existence of Mpox remain a possibility due to high CT values obtained from the specimen. Shotgun metagenomic sequencing (mNGS) successfully recovered sequences from VZV but it failed to generate any MPXV consensus sequence. With proper clinical evaluation and utilization of appropriate diagnostic tests, we were able to diagnose the first Filipino patient with a possible Mpox and varicella zoster virus co-infection. This report recommends to healthcare professionals to always diligently review laboratory results to help determine if there is a need for re-sampling and re-testing. Future studies on the possible mechanisms responsible for the presence of both Mpox and varicella zoster virus are also vital in the further understanding and surveillance of the disease.

## Data Availability

The original contributions presented in the study are included in the article/Supplementary materials, further inquiries can be directed to the corresponding author.
