# Supplementary material for: Clinical presentation and molecular diagnosis of a possible Mpox virus and Varicella zoster virus co-infection in an adult immunocompetent Filipino: a case report
Source: Front Public Health. 2024 Nov 21;12:1387636. doi: 10.3389/fpubh.2024.1387636 (PMC11617555; doi:10.3389/fpubh.2024.1387636)
Supplement: Supplementary file 1 [file Data_Sheet_1.pdf]

Clinical Presentation and Molecular Diagnosis of a Possible Mpox Virus and Varicella Zoster Virus Co-infection in an Adult Immunocompetent Filipino: A Case Report (Supplementary File)

April Keith Balingit[1†], Phoebe Grace Grande[1†], Amalea Dulcene Nicolasora[1], Francisco Gerardo Polotan[1], Roslind Anne Pantoni[1], Miguel Francisco Abulencia[1], Maria Yna Joyce Chu[1], Nicole Rivera[1], Marie Socouer Oblepias[1], Jemelyn Garcia[1]

[1]Research Institute for Tropical Medicine, Muntinlupa City, Philippines
[†]First authorship: These authors share first authorship

* Correspondence:
Phoebe Grace B. Grande
phoebe.grande@ritm.gov.ph

Table 1S. Primers and probes used for Mpox qPCR (12)

Primer/ Probe Name	Sequence 5' to 3'	Assay Name
G2R_G Forward Primer	5'- GGAAAATGTAAAGACAACGAATACAG-3'	G2R_G (Mpox Screening)
G2R_G Reverse Primer	5'- GCTATCACATAATCTGGAAGCGTA-3'	
G2R_G Probe	5' FAM-AAGCCGTAATCTATGTTGTCTATCGTGTCC-3' BHQ1	
G2R_WA Forward Primer	5'- CACACCGTCTCTTCCACAGA-3'	G2R_WA (Mpox Clade II)
G2R_WA Reverse Primer	5'- GATACAGGTTAATTTCCACATCG-3'	
G2R_WA Probe	FAM 5'-AACCCGTCGTAACCAGCAATACATTT-3' BHQ1	
C3L Forward Primer	5'- TGTCTACCTGGATACAGAAAGCAA-3'	C3L (Mpox Clade I)
C3L Reverse Primer	5'- GGCATCTCCGTTTAATACATTGAT-3'	
C3L Probe	5' FAM-CCCATATATGCTAAATGTACCGGTACCGGA-3' BHQ1	

Table 2S. Mpox RT-PCR result with mean cycle threshold (Ct) values

**Target**	**Sample**	**CT Value**	**Mean CT Value**	**Result**
G2R_G	Lesion surface dry swab MPOX22-00061DSA	35.06	36.20	Mpox Viral DNA Detected
	Lesion roof MPOX22-00061RA	37.33		
G2R_WA	Lesion surface dry swab MPOX22-00061DSA	35.08	35.62	Mpox Clade II** Viral DNA Detected
	Lesion roof MPOX22-00061RA	36.16		
C3L	Lesion surface dry swab MPOX22-00061DSA	Mpox Clade I* Viral DNA Not Detected		
	Lesion roof MPOX22-00061RA			

*previously named as Congo Basin clade
**previously named as West African clade
